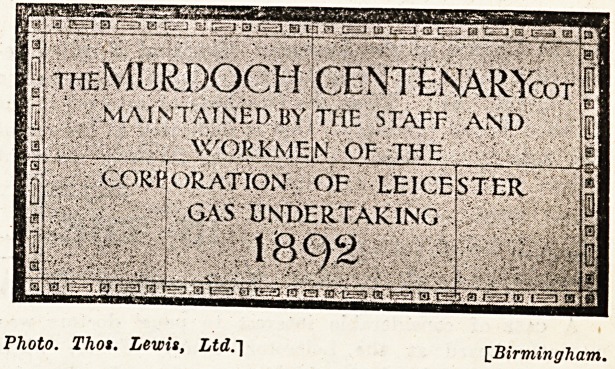# Endowed Beds with a History

**Published:** 1915-09-11

**Authors:** 


### ENDOWED BEDS WITH A HISTORY.

### II.—In the Provincial and Scotch Hospitals.

(FROM A CORRESPONDENT.)

The Cheltenham General Hospital has a bed with some curious directions attached to its endowment. The endower, Mr. H. W. Stubbin, directed in his will that "such bed shall not be occupied by an anti-vaccinator, nor by any relative, servant, or connection of a professed or suspected anti-vaccinator." It is to be hoped that the candidate for the bed does not die whilst these inquiries are gone into!

### The Result of a Chance Letter.

The following incident shows the value of writing for money, even if there is no reason to believe that it will come to anything. The secretary of the Guest Hospital, Dudley, saw an announcement in a local paper of a gift to a Primitive Methodist chapel by a gentleman at Sutton Coldfield. He wrote to him at a venture, but heard nothing for two months, when he received £500 and a gift of furniture, books, paintings, etc. Later, he wrote asking the secretary to go and see him. In August 1909 he died, and left the hospital the whole of his estate, valued at £84,000. The net result was over £70,000. Whether any of this money went to the endowment of beds I do not know, but thought it worth recording as showing the result of a chance letter.

*Photo. Thos. Lewis, Ltd.*] [*Birmingham.*

### A Link with the Navy at Leicester.

Leicester Royal Infirmary has two cots and one bed of note. Perhaps the most interesting of the former, having reference to the times we live in, is the "Peter" cot, maintained by Lady Beatty, named after Peter, the elder child of Sir David and Lady Beatty, who, his father and mother are good enough to say, was nursed back to health by one of the honorary staff of the hospital and one of the nursing sisters, after a very serious illness, in their Leicestershire seat.

The "Murdoch Centenary" cot is maintained by the employees of the local gas undertaking to perpetuate the name of William Murdoch. A bed is in process of endowment by the Leicester Football Club. This is the Rugby club associated with the town of Leicester, and I have an idea that it is the first bed of the kind endowed by a Rugby football club. The club every year sets aside a special match for the infirmary, and has in this way contributed over £1,600 to the funds of the infirmary.

### Birthday Beds.

The Blackburn and East Lancashire Infirmary have three beds endowed to commemorate various birthdays. Two are endowed as thankofferings for attaining a sixtieth and sixty-fifth birthday respectively. The third, the "Thomas and Margery Critchly," bed was endowed by the latter on the occasion of her eightieth birthday, April 15, 1915. The Royal Berkshire Hospital, Reading, and the Jenny Lind Infirmary for Children have a bed and cot respectively endowed in memory of officers killed in action in France.

### Some Royal Cots.

The Royal Alexandra Hospital, Rhyl, has a "Princess Mary" cot, given by the county of Shropshire in 1893 as a wedding gift to the Queen (then Duchess of York) on her marriage to our present King when Duke of York. The cot was to be called the "Princess Mary Shropshire Free" cot at the special request of the donors. Children from Shropshire have the prior claim, and all applications for it are made to the Queen.

### "Tiny Tim" and Children's Cots.

Another Royal cot, or rather bed, is at the Richmond Hospital. This was endowed by Her Majesty the Queen with a gift of £700 collected in Richmond and neighbourhood on the occasion of Her Majesty's Coronation. It is known as the "Queen Mary Coronation" bed.

*Photo. Thos. Lewis, Ltd.*] [*Birmingham.*

The Nottingham Children's Hospital contains a cot that should appeal to all children-lovers. The "Tom Herring Bingham" cot was endowed in 1893 in memory of Tom Herring Bingham, aged fifteen, who was drowned in heroically attempting to save the life of Cecily Frances Barber.

The Royal Alexandra Hospital, Rhyl, has also five cots endowed under the will of the late Mrs. Maud Hand, in memory of her only son, who died under very sad circumstances. The cots provide for five convalescent children resident in Staffordshire, and, failing such children, for those in any part of England and Wales.

The provinces as well as London possess a "Tiny Tim" cot, at the Royal Portsmouth Hospital, which is endowed by the members of the Portsmouth Dickens Fellowship.

The value of workmen's contributions to hospitals, both directly and indirectly, is illustrated by the "Workmen's" bed in the Royal Infirmary, Sunderland. This bed was endowed in 1914 by the Infirmary Ladies' Guild, in appreciation of the constant support given by the workmen of the district to the infirmary.

### Jubilees and Golden Weddings.

The Hospital for Women and Children, Leeds, has a bed endowed in memory of twenty-five happy years of married life, and a cot endowed to mark the jubilee of a local vicar. The "old girls" of the Maidstone Grammar School maintain a cot in the children's ward of the Maidstone General Hospital.

Alderman Mathias recently visited King Edward VII.'s Hospital, Cardiff, to see the patients, amongst whom were several wounded soldiers. He was so much impressed by all he saw that, as stated in The Hospital at the time, before leaving he handed to the secretary a cheque for 2,000 guineas to endow two beds in the "Coronation" ward—one in memory of his wife and the other in memory of his son. He wished his gift to date from May 25, as that would have been his golden wedding day. Hospital secretaries would doubtless welcome more visitors of a like kind, and may remember that others may desire to commemorate silver or golden wedding days by similar endowments.

The Gift of a Girls' School.

Some years ago the pupils of a girls' school determined to start a fund for the purpose of endowing a cot in the children's ward of the Huddersfield Royal Infirmary, and at each annual distribution of prizes they have held a sale of work. They have at length achieved their object, and £500 has been paid over to the treasurer, and a tablet, suitably inscribed, has been placed over the cot selected. At the Royal Isle of Wight County Hospital, Ryde, the "Mothers'" cot is in process of endowment by different mothers in the island.

A Pageant Bed.

The Council of the Lancaster Historical Pageant endowed a bed in the Royal Infirmary, to be known as the "Pageant" bed. There are two beds of interest at the Preston Royal Infirmary. One is dedicated in honour of the memory of Basil C. Middleton, formerly assistant house surgeon of this infirmary, who died on July 20, 1901, from scarlet fever, contracted in the exercise of his duties as a member of the staff. The other is the "St. John Ambulance Brigade Preston Corps" bed, dedicated to the memory of three members of the above corps who died whilst on active service during the war in South Africa.

"Alice in Wonderland."

Lovers of the works of "Lewis Carroll" will be interested to know that there is a cot endowed in memory of him at the Western Infirmary, Glasgow, known as the "Alice in Wonderland" cot.

The late Mr. Murdoch in 1897 left £1,000 to Chalmers Hospital, Edinburgh, for the upkeep of a bed; when it is vacant the officers of the Northern Lighthouse Service and their wives and families have preference of occupancy. The children in the various Sunday schools throughout the Church of Scotland have endowed a cot in the Deaconess Hospital, Edinburgh, known as the "Morning Rays" cot, through the Editor of *Morning Rays*, the children's magazine.

A lieutenant in the Gordon Highlanders, who was attached to the Imperial Light Infantry in the South African War, and was killed in action at Spion Kop, left funds to endow a bed in the Turner Memorial Hospital, Keith.

An Accident Bed.

The citizens of Leith are going to endow a bed or beds in the local hospital in memory of the men of the 7th Royal Scots who were killed in the Gretna railway accident just recently.

Two beds have been endowed at the Victoria Hospital, Helensburgh, by Mrs. Drew in name of her husband and herself, she being a centenarian at her death.

## Figures and Tables

**Figure f1:**
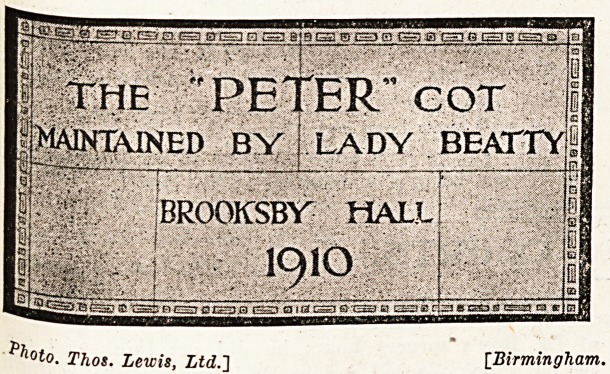


**Figure f2:**